# Sitagliptin activates the p62–Keap1–Nrf2 signalling pathway to alleviate oxidative stress and excessive autophagy in severe acute pancreatitis-related acute lung injury

**DOI:** 10.1038/s41419-021-04227-0

**Published:** 2021-10-11

**Authors:** Lingming Kong, Jie Deng, Xiang Zhou, Binbin Cai, Baofu Zhang, Xiaohu Chen, Zongjing Chen, Weiming Wang

**Affiliations:** 1 Department of Hepatopancreatobiliary Surgery, The First Affiliated Hospital of Wenzhou Medical University, Wenzhou, Zhejiang China; 2 Key Laboratory of Diagnosis and Treatment of Severe Hepato-Pancreatic Diseases of Zhejiang Province, The First Affiliated Hospital, Wenzhou Medical University, Wenzhou, China; 3 Department of Breast Surgery, The First Affiliated Hospital of Wenzhou Medical University, Wenzhou, Zhejiang China; 4 Department of Pathology, The First Affiliated Hospital of Wenzhou Medical University, Wenzhou, China

**Keywords:** Macroautophagy, Acute pancreatitis

## Abstract

Acute lung injury (ALI) is a complication of severe acute pancreatitis (SAP). Sitagliptin (SIT) is a DPP4 inhibitor that exerts anti-inflammatory and antioxidant effects; however, its mechanism of action in SAP-ALI remains unclear. In this study, we investigated the effects of SIT on SAP-ALI and the specific pathways involved in SAP-induced lung inflammation, including oxidative stress, autophagy, and p62–Kelch-like ECH-associated protein 1 (Keap1)–NF-E2-related factor 2 (Nrf2) signalling pathways. Nrf2 knockout (Nrf2^−/−^) and wild-type (WT) mice were pre-treated with SIT (100 mg/kg), followed by caerulein and lipopolysaccharide (LPS) administration to induce pancreatic and lung injury. BEAS-2B cells were transfected with siRNA-Nrf2 and treated with LPS, and the changes in inflammation, reactive oxygen species (ROS) levels, and autophagy were measured. SIT reduced histological damage, oedema, and myeloperoxidase activity in the lung, decreased the expression of pro-inflammatory cytokines, and inhibited excessive autophagy and ROS production via the activation of the p62–Keap1–Nrf2 signalling pathway and promotion of the nuclear translocation of Nrf2. In Nrf2-knockout mice, the anti-inflammatory effect of SIT was reduced, resulting in ROS accumulation and excessive autophagy. In BEAS-2B cells, LPS induced ROS production and activated autophagy, further enhanced by Nrf2 knockdown. This study demonstrates that SIT reduces SAP-ALI-associated oxidative stress and excessive autophagy through the p62–Keap1–Nrf2 signalling pathway and nuclear translocation of Nrf2, suggesting its therapeutic potential in SAP-ALI.

## Introduction

Acute pancreatitis is a destructive pancreatic inflammatory disease with a global incidence of 34 cases per 100,000 individuals annually, which is increasing worldwide [1, 2]. Common causes of acute pancreatitis trigger pathological cellular responses and abnormal organelle function, ultimately leading to pancreatic acinar cell death with local and systemic inflammation [3]. Many patients develop mild pancreatitis, but 20% develop aggravated severe acute pancreatitis (SAP), characterised by persistent (>48 h) organ failure [4, 5]. Acute lung injury (ALI) and acute respiratory distress syndrome are the dominant death-associated factors in patients with early-stage acute pancreatitis [6]. Although our understanding of the mechanisms underlying SAP and associated ALI is increasing, there are currently no straightforward recommendations for the effective treatment of this disease.

Dipeptidyl peptidase-4 (DPP4) is a type-II transmembrane glycoprotein with a short cytoplasmic tail of six amino acids. Its active state is a dimer, and the molecular weight of the monomer is 110 kDa [7]. DPP4 is highly expressed in many lung diseases [8–11]. In 2006, sitagliptin (SIT) was authorised by the U.S. Food and Drug Administration and became the first clinically used DPP4 inhibitor (DPP4i) to treat type 2 diabetes [12]. In an SAP mouse model, SIT reduces the expression of DPP4 in the intestine and inhibits oxidative stress and inflammation through the Nrf2–NF-κB pathway [13]. To the best of our knowledge, the function of SIT in SAP-ALI and its latent mechanism have not been studied. Studies have partially unveiled the relationship between DPP4i and autophagy [14, 15].

Autophagy refers to the self-digestion process of cells using lysosomes to degrade macromolecular substances and organelles that are destroyed, denatured, senescent, and under the influence of external environmental factors. The pathogenesis of acute pancreatitis is complex, and important cellular and molecular events include impaired autophagy. Autophagy regulates cell survival or death in diverse cell types, environments, and stress stimuli [16]. Excess autophagy results in autophagic cell death [17]. Furthermore, autophagy disorders promote inflammatory responses in the pancreas [18]. Under pathological conditions such as ischaemia/reperfusion or tumour hypoxia, oxidative stress and autophagy are induced in response to excessive ROS accumulation. Conversely, autophagy can alleviate oxidative damage by degrading or phagocytising oxidative substances [19, 20]. Reactive oxygen species (ROS) participate in the inflammatory cascade that mediates inflammatory cell recruitment and tissue damage [21, 22]. Oxidative stress and ROS are involved in SAP-related pancreatic acinar cell damage [23]. The Kelch-like ECH-associated protein 1 (Keap1)–NF-E2-related factor 2 (Nrf2)-antioxidant response element (ARE) system is the main reaction mechanism to anti-oxidative stress damage. The absence or disrupted activation of Nrf2 aggravates the body’s oxidative stress state and disrupts normal cellular redox homoeostasis, leading to cell dysfunction and even death [24]. p62/SQSTM-1 is a multifunctional multifaceted adaptor protein, the main function of which is to carry ubiquitinated proteins to the proteasome for degradation. p62 has a regulatory effect on Keap1 [25]. The p62–Keap1–Nrf2 signalling pathway participates in the regulation of poisoning, human hepatocellular carcinomas, apoptosis, and autophagy [26, 27]. Therefore, the mechanism underlying autophagy changes in pancreatitis-related lung injury and relationship between autophagy and Nrf2 pathway warrant further studies.

In this study, we aimed to verify whether SIT has a protective effect against SAP-related ALI. We hypothesised that the protective action of SIT occurs through p62–Keap1–Nrf2 signalling pathway upregulation to reduce ROS levels and regulate autophagy. Whether nuclear translocation of Nrf2 occurs in SAP-ALI, the state change of Nrf2 after inflammation and SIT pre-treatment, and the relationship among this pathway, autophagy, and DPP4 are worthy of further investigation.

## Materials and methods

### Materials

Sitagliptin (SIT; cat. No. HY-13749) and caerulein (HY-A0190) were obtained from MedChemExpress (Princeton, NJ, USA). Lipopolysaccharide (LPS; from *E. coli* O127: B8) was purchased from Sigma (St. Louis, MO, USA). Antibodies against LC3-ll (4108), Beclin1 (3738), Atg5 (2630), and NQO1 (62262) were purchased from Cell Signaling Technology Inc. (Beverly, MA, USA); MPO (ab9535), 4-HNE (ab46545), DPP4 (ab187048), and Lamin B1(ab16048) were purchased from Abcam Inc. (Cambridge, MA, USA); and β-actin (66009-1-Ig), Nrf2 (16396-1-AP), Keap1 (10503-2-AP), Ho-1 (10701-1-AP), and p62 (18420-1-AP) were purchased from Proteintech Inc. (Shanghai, China).

### Animal experiment protocol

C57BL/6 wild-type (WT) mice were purchased from the Laboratory Animal Center of Wenzhou Medical University (Wenzhou, China). Nrf2-knockout (Nrf2^−/−^) mice with the C57BL background were obtained from the Experimental Animal Centre of Nanjing Medical University (Jiangsu, China). Experts’ reports of knockout effects are provided as Supplementary Materials. In brief, 6-week-old male mice weighing 20–25 g were used. The experiments were performed in accordance with the guidelines of the Institutional Animal Committee of Wenzhou Medical University. Caerulein pancreatitis was induced as previously described [13, 28, 29]. WT and Nrf2^−/−^ mice were randomly distributed into four experimental groups (*n* = 8/group): control group (CON)—0.9% saline; SAP group (SAP)—caerulein (50 μg/kg, 7 times) + LPS (10 mg/kg); SIT group (SIT)—SIT (100 mg/kg); and SAP + SIT group (SAP + SIT)—SIT (100 mg/kg) + caerulein (50 μg/kg, 7 times) + LPS (10 mg/kg). SIT was injected intraperitoneally 1 h before the first caerulein injection. Surgical procedures were performed under deep intraperitoneal anaesthesia after xylazine (5 mg/kg) premedication and intraperitoneal injection of ketamine (100 mg/kg). All animals were euthanised 24 h after the last drug injection.

### Histopathological analysis

Pancreatic and pulmonary tissues were fixed in 4% paraformaldehyde for 48 h, dehydrated in an ascending gradient alcohol solution, embedded in paraffin wax, and sectioned at 4-μm-thick. Sections were deparaffinised twice for 20 min in xylene, rehydrated through a descending graded ethanol solution for 5 min each, and rinsed in deionised water for 5 min. The sections were stained with haematoxylin and eosin (HE) (G1120; Solarbio, Shanghai, China). Pathological alterations in the pancreas and lungs were observed under a light microscope (Leica, Jena, Germany) at ×200 magnification. Subsequently, pancreatic pathological and lung injuries were scored following a previous description [30, 31]. Each section was partitioned into five equal parts and scored. Scoring was performed by two researchers independently according to standard protocols, and discrepancies were arbitrated by a third investigator.

### Lung wet-to-dry weight (W/D) ratio

An electronic scale was used to weigh the tissue samples that were removed from the mice. Subsequently, the samples were gradually dried in a 70 °C oven until they were stabilised at a dry weight after 48 h. The lung W/D ratio was calculated using the following formula: (wet weight−dry weight)/dry weight.

### Immunohistochemical (IHC) analysis

To examine the expression of MPO and 4-HNE in the lung tissue samples, 4-μm-thick sections of formalin-fixed, paraffin-embedded lung tissues were used for IHC analysis. The sections were deparaffinised using xylene and rehydrated in five graded concentrations of alcohol solutions. For antigen repair, the sections were heated in 0.01 M citrate-hydrochloric acid for 15 min at full strength using a microwave. After blocking non-specific proteins with 5% bovine serum albumin at 37 °C for 1.5 h, MPO antibody and 4-HNE antibody were applied to sections and incubated at 4 °C overnight. After incubation with the peroxidase-conjugated secondary antibodies, staining with diaminobenzidine (DAB, P0202, Beyotime, Shanghai, China) and counterstaining with haematoxylin were performed. Negative controls were replaced with rabbit IgG rather than primary antibodies. The sections were observed under a light microscope at ×200 magnification (Leica, Jena, Germany). Microscopic images were assessed using ImageJ software to determine the integral optical density (IOD) and the area of each picture. The average optical density (AOD) was calculated using the following formula: AOD = IOD/area.

### Immunofluorescence

Sections were prepared as described in the IHC analysis. For immunostaining, the sections were incubated overnight with primary antibodies against LC3-ll at 4 °C. After washing with phosphate-buffered saline (PBS) three times, sections were incubated with an Alexa Fluor 488-conjugated anti-rabbit secondary antibody (33106ES60, YEASEN, Shanghai, China) in the dark for 30 min. The nuclei were stained with 4′,6-diamidino-2-phenylindole (36308ES20; YEASEN) for 5 min in the dark. Images of the lung tissue were visualised using Leica TCS SP8 (Leica Microsystems, CMS GmbH, Wetzlar, Germany).

### Transmission electron microscopy

Small pieces of lung tissue were placed on a slide and 2.5% glutaraldehyde was added. Subsequently, the slide was gently pressed against another slide to remove bubbles. Fresh lung tissue was pre-fixed in 2.5% glutaraldehyde for 2 h, rinsed twice with PBS, and post-fixed in 0.01 g/mL osmium tetroxide for 1 h. After fixation, lung tissues were dehydrated in an ascending gradient acetone solution and embedded in epoxy resin. Ultrathin sections were stained with uranyl acetate and lead citrate and examined under a transmission electron microscope (TEM) (Hitachi H-7500; Hitachi, Tokyo, Japan).

### Small interfering RNA (siRNA) transfection

siRNAs targeting Nrf2 (si-Nrf2) were obtained from GenePharma (Shanghai, China). Their specific sequences were 5′-CCGGCAUUUCACUAAACACAATT-3′ (si-Nrf2-1) and 5′-GCAGCAAACAAGAGAUGGCAATT‐3′ (si-Nrf2-2). The control group was transfected with NC-siRNA, the specific sequence of which was 5′-UUCUCCGAACGUGUCACGUTT‐3′. BEAS-2B cells were transfected with 50 nM siRNA using Lipofectamine 3000 (Invitrogen, Carlsbad, CA, USA) for 48 h. The cells were collected at 48 h, and knockdown efficiency was confirmed by quantitative real-time PCR (qRT-PCR).

### Cell culture

Human lung epithelial BEAS-2B cells were obtained from the Cell Bank of the Chinese Academy of Sciences (Shanghai, China) and cultured in Dulbecco’s modified Eagle’s medium supplemented with 10% foetal-bovine serum (Gibco, Carlsbad, CA, USA), 100 U/mL streptomycin, and 100 μg/mL penicillin. The cells were maintained at 37 °C with 5% CO_2_. Experiments were performed when BEAS-2B cells reached 80–90% confluence. We used LPS (1 μg/mL) to establish a stabilised inflammatory model in BEAS-2B cells [32, 33]. The cells were divided into four groups: (1) the cells were transfected with 50 nM NC-siRNA (CON/NC-siRNA); (2) LPS (1 μg/mL) was added to the cells after transfection with 50 nM NC-siRNA (LPS/NC-siRNA:); (3) the cells were transfected with 50 nM si-Nrf2 (CON/si-Nrf2), and (4) LPS (1 μg/mL) was added to the cells after transfection with 50 nM si-Nrf2 (LPS/si-Nrf2). We used two siRNAs targeting Nrf2 to rule out off-target effects.

### Flow cytometry

Intracellular ROS levels in BEAS-2B cells were tested using an oxidation-sensitive dihydroethidium (DHE) fluorescent probe (S0063, Beyotime). After incubating Nrf2 siRNA with BEAS-2B cells for 24 h, the cells were treated with LPS (1 µg/mL) for 6 h [34]. The cells were then incubated with DHE for 20 min at 37 °C, rinsed with serum-free medium three times to remove excess DHE, collected by trypsinisation, and resuspended in PBS. The cells were analysed using a flow cytometer (FACSCalibur, BD, Franklin Lakes, NJ, USA). Flow cytometry data were analysed using FlowJo software (Tree Star, San Carlos, CA, USA).

### Quantitative real-time PCR

Using the TRIzol Reagent (15596026; Thermo Fisher Scientific, Carlsbad, CA, USA), RNA was extracted from the lung tissues and BEAS-2B cells. To quantify the amount of mRNA, cDNA was generated from 1 μg of RNA using the RevertAid First Strand cDNA Synthesis Kit (Fermentas, Milan, Italy), with the final volume of 20 μL (K1622; Thermo Fisher Scientific). Next, qRT-PCR was performed using SYBR Green Supermix with ROX (A25742; Thermo Fisher Scientific, Hertfordshire, UK) and a PCR detection system (7500fast; Applied Biosystems, Foster City, CA, USA). The results were statistically analysed using the 2^−ΔΔCt^ method, with β-actin as an internal normalisation control. All primer sequences are shown in Table 1.

**Table 1:** Sequences of the primers used for quantitative real-time PCR.

	Gene	Forward primer (5′–3′)	Reverse primer (5′–3′)
Mouse	β-Actin	GTGCTATGTTGCTCTAGACTTCG	ATGCCACAGGATTCCATACC
	IL-6	TCTGCTCTGGAGCCCACCAAG	CCAGCATCAGTCCCAAGAAGGC
	TNF-α	AAGGGAGAGTGGTCAGGTTGCC	TGTGAGGAAGGCTGTGCATTGC
	IL-1β	GCAGCAGCACATCAACAAGAGC	AGGTCCACGGGAAAGACACAGG
BEAS-2B cell	β-Actin	CACGATGGAGGGGCCGGACTCATC	TAAAGACCTCTATGCCAACACAGT
	Nrf2	TCCAAGTCCAGAAGCCAAACTGAC	GGAGAGGATGCTGCTGAAGGAATC
	IL-6	CTGCAAGAGACTTCCATCCAG	AGTGGTATAGACAGGTCTGTTGG
	IL-1β	GAAATGCCACCTTTTGACAGTG	TGGATGCTCTCATCAGGACAG
	TNF-α	CAGGCGGTGCCTATGTCTC	CGATCACCCCGAAGTTCAGTAG

### Western blotting

The total protein content in the lung tissues and BEAS-2B cells were extracted using RIPA buffer (P0013B; Beyotime Biotechnology, Shanghai, China), with the addition of phenylmethanesulfonylfluoride (ST506; Beyotime Biotechnology) and PhosSTOP (Roche, Basel, Switzerland). Nuclear and cytosol extracts from the lung tissue were prepared using the Nuclear Extraction Kit (Solarbio, R0050, Shanghai, China). Protein concentrations were determined using a bicinchoninic acid assay kit (P0012; Beyotime Biotechnology). After denaturation, the same amounts of protein from all samples were electrophoresed on SDS-PAGE gels and transferred to a 0.45-μm polyvinylidene fluoride membrane (Millipore, Billerica, MA, USA). The membranes were blocked with 5% skim milk for 2 h at room temperature. The membranes were probed with primary antibodies at 4 °C overnight, followed by incubation with the corresponding secondary antibodies (Biosharp, China) 1 h at 37 °C. Finally, immunoreactive bands were visualised by enhanced chemiluminescence and quantified by densitometry using VisionWorks imaging software (Eastman Kodak Company, Rochester, NY, USA).

### Statistical analysis

Values are presented as mean ± SEM. Statistical analysis was performed using GraphPad Prism 8.0 (GraphPad Software, San Diego, CA, USA). One-way analysis of variance was used to determine differences among three or more groups. Student’s *t-*test (two tailed) was used to compare differences between groups. The results were calculated using data from three independent experiments. *P* values are denoted as *P < 0.05 and **P < 0.01 in all figures.

## Results

### Sitagliptin attenuates SAP inflammation and the associated ALI in mice

**Fig. 1:**
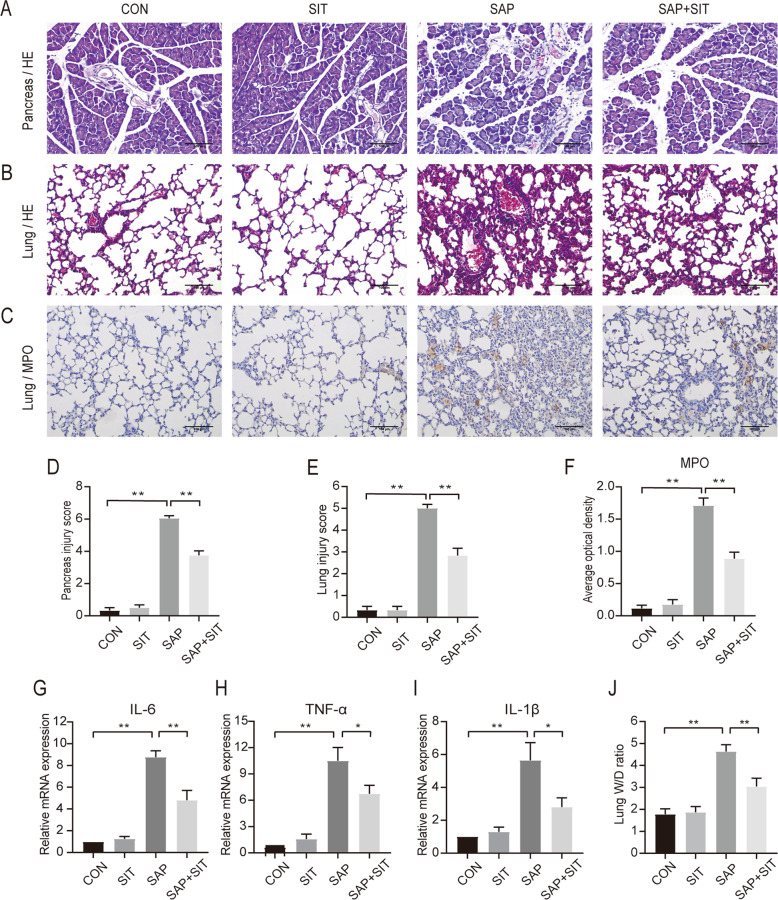
Sitagliptin inhibited SAP and SAP-ALI in mice. **A**, **B** Representative H&E staining iamge of the pancreas and lung tissue (×200). **C** Immunohistochemical staining of MPO revealed inflammation in the lung (×200). **D** The pancreatitis score. **E** The lung injury score. Slides were evaluated by two independent investigators in a blinded manner. **F** the quantification of MPO Immunohistochemical staining using the following formula: AOD = IOD/area. **G–I** Real-time PCR results of IL-6,TNF-α and IL-1β in different groups. **J** The lung W/D ratio. *P < 0.01 and **P < 0.05. Data are presented as mean ± standard error of the mean (SEM) (*n* = 6).

The SAP group showed significantly increased interstitial tissue oedema, inflammatory cell infiltration, and pancreatic acinar cell vacuolation or necrosis. No apparent pathological changes were noted in the CON and SIT groups. Compared with the SAP group, the SAP + SIT group exhibited normal pancreatic tissue structure (Fig. 1A). The CON and SIT groups showed normal pulmonary architecture (Fig. 1B). The SAP group showed noticeable morphological changes, including alveolar septum thickening, alveolar oedema or collapse, blood vessel hyperaemia, and inflammatory cell infiltration in the pulmonary architecture. In contrast, the SIT treatment group showed a noticeable improvement. The severity of pancreatitis was quantified by measuring the pathology score. The histological score of the pancreas was lower in the SAP + SIT group than in the SAP group (Fig. 1D). Detailed histological scores of pancreatic damages, oedema, inflammatory cell infiltration, and acinar cell necrosis are shown in Supplementary Fig. 1a–c. Lung injury scores were comparable to pancreatic injury scores (Fig. 1E). MPO in the lung tissue is a specific marker that reflects inflammation and neutrophil infiltration into damaged tissue [35]. Representative images of MPO-stained lung tissue sections are shown in Fig. 1C. The IHC images in Fig. 1F indicate that the MPO level considerably increased in the SAP group. When treated with SIT, mice showed alleviated damage with weaker MPO staining. interleukin-6 (*IL-6*), tumour necrosis factor*-*α (*TNF-α*), and *IL-1β* levels were significantly higher in the SAP group than in the CON and SIT groups (Fig. 1G–I). These pro-inflammatory cytokine levels were reduced by SIT treatment. Consistent with these results, the lung W/D ratio was considerably elevated in the inflammation group, whereas the SIT treatment reduced caerulein- and LPS-induced pulmonary oedema (Fig. 1J).

### Sitagliptin downregulates the autophagy level in SAP-ALI

**Fig. 2:**
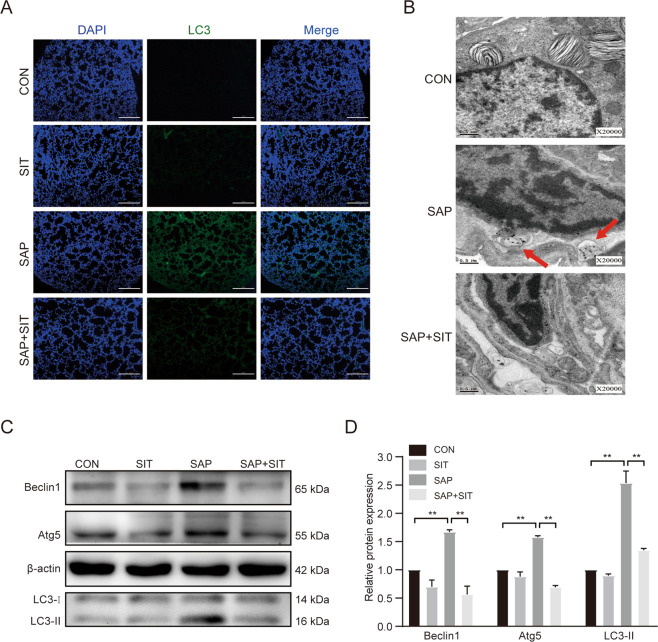
Sitagliptin downregulated autophagy level in SAP-ALI. **A** Immunofluorescence staining for LC3-II in the lung (×200). **B** The representative TEM photo of the lung tissue. Red arrows: autophagic vacuoles (AVs). **C**, **D** Western blotting results and analysis of Beclin1, Atg5, and LC3-ll in different groups. Beclin1 and Atg5 versus β-actin, LC3-II versus LC3-I. **P < 0.05. Data are presented as mean ± standard error of the mean (SEM) (*n* = 6).

Immunofluorescence staining revealed that the LC3-II level increased in the SAP group, whereas the LC3-II level reduced in SIT pre-treated mice (Fig. 2A). The autophagic vacuoles (AVs) were more evident in the SAP group than in other groups (Fig. 2B). The expression of Beclin1, Atg5, and LC3-ll was higher in the SAP group than in the CON and SIT groups (Fig. 2C, D). The expression of these proteins was reduced by SIT.

### Sitagliptin reduces ROS levels and activates the P62–Keap1–Nrf2 signalling pathway

**Fig. 3:**
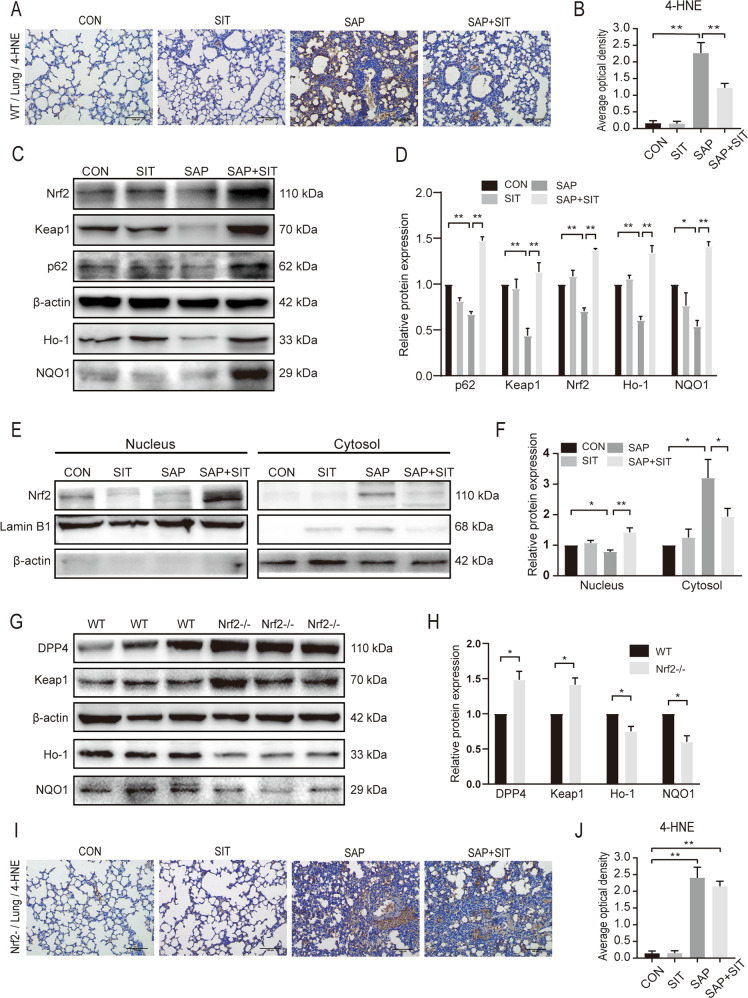
Sitagliptin reduced ROS levels and activated the p62–Keap1–Nrf2 signalling pathway. **A**, **B** In WT mice, immunohistochemical staining and quantification of 4-HNE revealed ROS in the lung. Quantification using the following formula: AOD = IOD/area. **C**, **D** Western blotting results and analysis of p62, Keap1, Nrf2, Ho-1, and NQO1 in different groups. **E**, **F** Western blotting results and analysis of Nrf2 in nucleus and cytosol. **G**, **H** Western blotting results and analysis of DPP4, Keap1, Ho-1, and NQO1 in the CON group of WT mice and Nrf2^−/−^ mice. **I**, **J** After Nrf2 deficiency, the immunohistochemical staining and quantification of 4-HNE in Nrf2^−/−^ mice. Quantification using the following formula: AOD = IOD/area. **P < 0.05. Data are presented as mean ± standard error of the mean (SEM) (*n* = 6).

The levels of 4-HNE were significantly higher in the SAP and SAP + SIT groups than in the Con and SIT groups (Fig. 3A). SIT reduced the level of 4-HNE compared with that in the SAP group (Fig. 3B). The expression of p62, Keap1, Nrf2, and the downstream targets, Ho-1 and NQO1, was lower in the SAP group than in the CON, SIT, and SAP + SIT groups (Fig. 3C, D). SIT, to some extent, promoted Nrf2 nuclear translocation (Fig. 3E). Additionally, the Nrf2 level was relatively high in the cytoplasm of the SAP group (Fig. 3F). The expression of DPP4 and Keap1 was higher in Nrf2^−/−^ mice than in WT mice (Fig. 3G, H). In contrast, the expression of NQO1 and Ho-1 was downregulated after Nrf2 knockout. The levels of 4-HNE in the SAP and SAP + SIT groups did not significantly differ after Nrf2 knockdown (Fig. 3I, J).

### Anti-inflammatory function of sitagliptin is counteracted in Nrf2-knockout mice

**Fig. 4:**
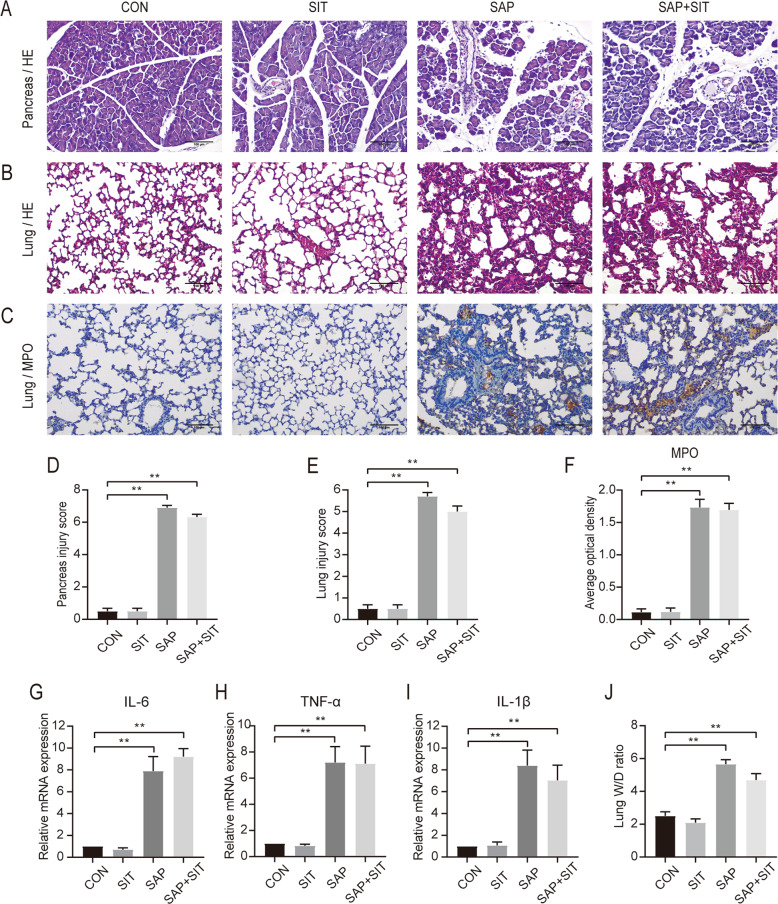
The protective effect of sitagliptin was significantly reduced in Nrf2^−/−^ mice. **A**, **B** Representative H&E staining iamge of the pancreas and lung tissue (×200). **C** Immunohistochemical staining of MPO revealed inflammation in the lung (×200). **D** The pancreatitis score. **E** The lung injury score. Slides were evaluated by two independent investigators in a blinded manner. **F** The quantification of MPO Immunohistochemical staining using the following formula: AOD = IOD/area. **G–I** Real-time PCR results of IL-6,TNF-α, and IL-1β in different groups. **J** The lung W/D ratio. **P < 0.05 versus the CON group. Data are presented as mean ± standard error of the mean (SEM) (*n* = 6).

In the Nrf2^−/−^ mice, H&E staining intensity and the corresponding pathology score histogram indicated that SIT treatment did not reduce the inflammation in pancreatic and lung tissues (Fig. 4A, B, D, E). Detailed histological scores of pancreatic damages, oedema, inflammatory cell infiltration, and acinar cell necrosis are shown in Supplementary Fig. 1d–f. In mice with Nrf2 deficiency, tissue concentrations of MPO were higher in the SAP group than in the CON and SIT groups, whereas SIT did not decrease the MPO concentration modulated by SAP (Fig. 4C, F). There were no evident changes in IL-6, TNF-α, and IL-1β levels between the treatment groups and the SAP group (Fig. 4G–I). The lung W/D ratios were consistent with these results (Fig. 4J). We also compared H&E staining intensity and the corresponding pathology scores between WT and Nrf2^−/−^ mice in the SAP group and found more severe inflammation in Nrf2-knockout mice (Supplementary Fig. 2a–d). Similarly, we compared the lung W/D ratio and found more severe pulmonary oedema in the knockout mice (Supplementary Fig. 2e). Our results showed that SIT-mediated inhibition of inflammation in the pancreas and lung was lower in Nrf2^−/−^ mice. Thus, the effect of SIT on SAP-related ALI might rely on Nrf2 activation.

### Nrf2 knockout could suppress the inhibitory effect of sitagliptin on autophagy in mice

**Fig. 5:**
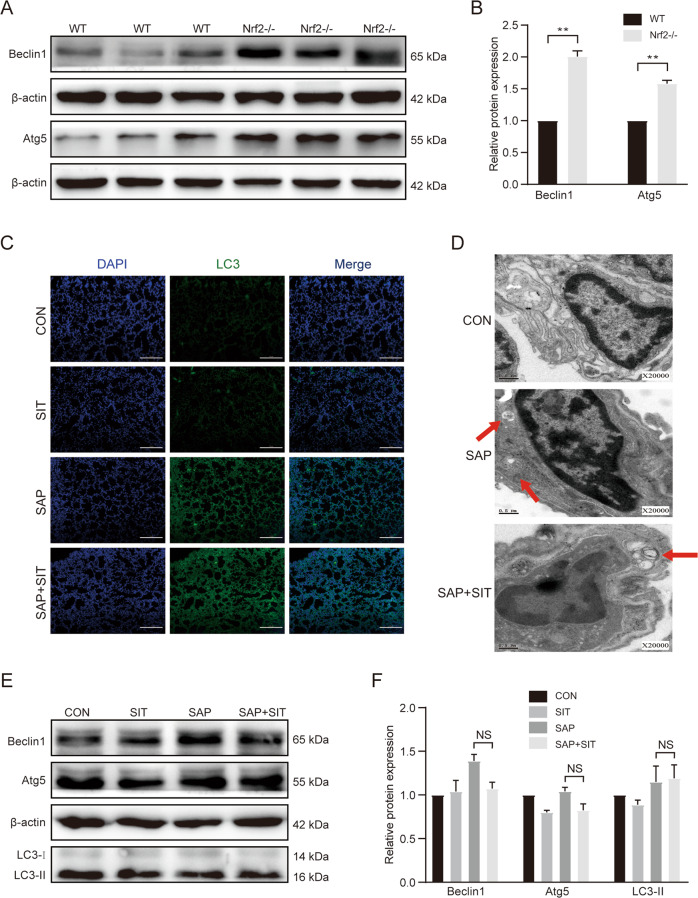
Knocking out Nrf2 partially abolished the inhibition of autophagy of Sitagliptin on mice. **A**, **B** Western blotting results and analysis of Beclin1 and Atg5 in the CON group of WT mice and Nrf2^−/−^ mice. **C** Immunofluorescence staining for LC3-II in the lung (×200). **D** The representative TEM photo of the lung tissue. Red arrows: autophagic vacuoles (AVs). **E**, **F** Western blotting results and analysis of Beclin1, Atg5, and LC3-II in different groups. Beclin1 and Atg5 versus β-actin, LC3-II versus LC3-I. **P < 0.05 versus the WT mice. Data are presented as mean ± standard error of the mean (SEM) (*n* = 6).

The expression of Beclin1 and Atg5 was higher in Nrf2^−/−^ mice than in WT mice (Fig. 5A, B). Immunofluorescence staining revealed that the LC3-ll level was increased in the SAP and SAP + SIT groups (Fig. 5C). TEM of the ultrastructure of lung tissues in Nrf2-knockout mice revealed that AVs were evident in both SAP and SAP + SIT groups (Fig. 5D). Protein levels were measured in lung tissue by western blotting; the expression levels of Beclin1, Atg5, and LC3-ll were not significantly different among the four groups. These protein levels were not reduced by SIT treatment (Fig. 5E, F).

### Nrf2 knockdown enhances autophagy in BEAS-2B cells

**Fig. 6:**
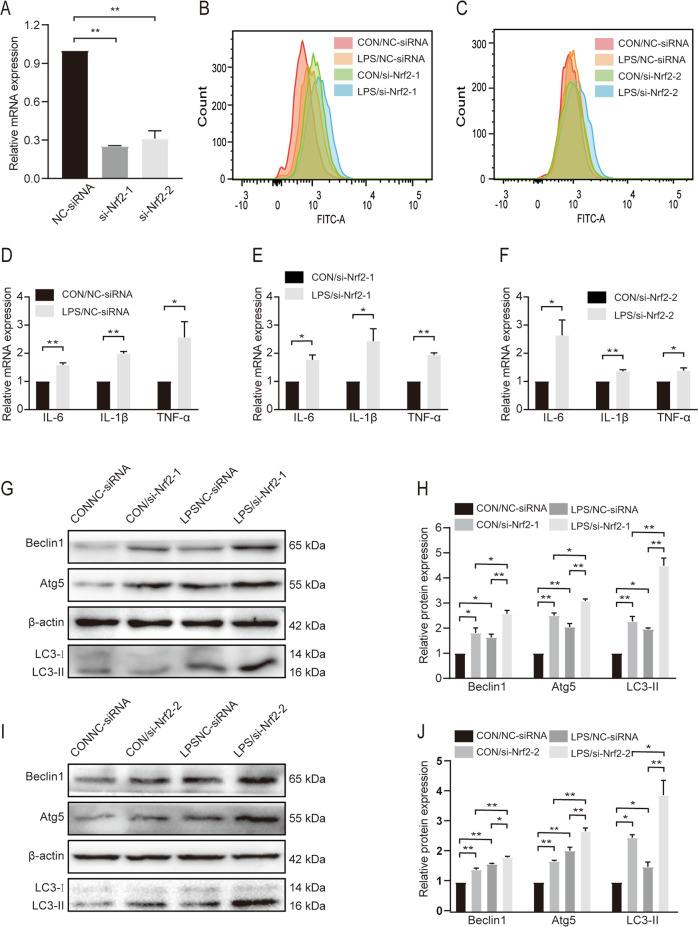
Knockdown of Nrf2 enhanced autophagy level in BEAS-2B cells. **A** qRT‐PCR results of Nrf2 expression in siRNA‐transduced cells. After 48 h transduction with siRNA, the cells were subjected to qRT‐PCR. **B**, **C** The levels of ROS were determined by flow cytometric analysis. **D–F** qRT-PCR results of IL-6, IL-1β, and TNF-α in different groups. **G–J** Western blotting results and analysis of Beclin1, Atg5, and LC3-II in different groups. Beclin1 and Atg5 versus β-actin, LC3-II versus LC3-I. *P < 0.01 and **P < 0.05. Data are presented as mean ± standard error of the mean (SEM) (*n* = 3).

The effects of Nrf2 knockdown were confirmed by qRT-PCR (Fig. 6A). The IL-6, IL-1β, and TNF-α levels increased 24 h after LPS treatment (Fig. 6D–F). ROS levels were higher in the LPS/NC-siRNA, CON/si-Nrf2, and LPS/si-Nrf2 groups than in the CON/NC-siRNA group (Fig. 6B, C). The ROS levels in the LPS/si-Nrf2 group were the highest among the four groups. To determine whether Nrf2 knockdown led to increased autophagy levels in BEAS-2B cells, we examined the expression of autophagy-related proteins. LPS promoted Beclin1, Atg5, and LC3-ll expression in BEAS-2B cells, and protein expression was further increased after Nrf2 knockdown (Fig. 5G–J).

## Discussion

DPP4, a membrane-bound aminopeptidase, is generally present in the plasma. It differentially regulates glucose homoeostasis and inflammation through its enzymatic activity and non-enzymatic immunomodulatory effects [36]. Considering reports of the high expression of DPP4 in lung diseases and anti-inflammatory function of DPP4i in vitro and in vivo, we speculated that DPP4i might be a potential therapeutic candidate for SAP-ALI [33, 37, 38]. Previously [13], we used ELISA to detect DPP4 in plasma, and showed that sitagliptin reduces the level of DPP4. Consistent with these reports, our findings demonstrate that SIT can reduce the inflammatory reaction in a mouse model of SAP stimulated by caerulein and LPS. Unexpectedly, our findings indicated that the level of autophagy-related proteins increased in the inflammatory state, and AVs were apparent in the SAP group under TEM. The level of autophagy-related proteins increased in the LPS-induced inflammation model of BEAS-2B cells. Sitagliptin-pre-treated mice showed a lower autophagy response. Therefore, SIT might be a valuable therapeutic drug for SAP-ALI, and its pharmacological mechanism is related to excessive autophagy.

Physiological levels of autophagy can maintain cell homoeostatic metabolism in response to environmental stimuli, but excessive or insufficient autophagy can cause diseases [39, 40]. Consistent with our results, some studies have indicated that endoplasmic reticulum stress and transitional autophagy inhibition can protect against LPS-induced ALI in vivo and in vitro [41–43]. Moreover, autophagy induction, thereby trypsinogen activation, can cause pancreatitis [44–46]. Therefore, autophagy is closely associated with the development of acute pancreatitis. p62 is a ubiquitin-bound autophagy receptor protein that links the Nrf2 pathway and autophagy [47]. Unexpectedly, after Nrf2 knockout, the autophagy response in mice was enhanced, and the therapeutic effect of SIT disappeared. A previous study confirmed that the anti-tumour effect of DPP4is in colorectal cancer leads to apoptosis and promotes cell cycle regulation by downregulating autophagy [15]. The inhibitory effect of SIT on autophagy is consistent with our results. Therefore, we speculate that autophagy overactivation aggravates the signs of SAP-ALI, and SIT inhibits inflammation and excessive autophagy through the Nrf2 pathway.

The Keap1–Nrf2–ARE pathway, the main defence system in cells, protects against oxidative damage and maintains homoeostasis. We compared H&E staining intensity and the corresponding pathology scores between WT and Nrf2^−/−^ mice in the SAP group. The change in histological damage in SAP mice with Nrf2 deficiency was limited. Although the pathology was not clear, we observed that the state of the mice was significantly different during modelling, and the activity of mice in the knockout SAP group was particularly poor. After knockout, the W/D ratio was significantly higher and blood inflammation was more severe. Consistent with our findings, Liu et al. [48] reported that administering an Nrf2 inhibitor alleviates the severity of AP in mice. p62 phosphorylation or abnormal accumulation significantly reinforces its binding to Keap1, thereby prompting the dissociation of Keap1 and Nrf2 [49–52]. Nrf2 is then transported to the nucleus, triggering the transcription of downstream antioxidant enzymes, such as HO-1 and NQO1, and protecting the cell from oxidative damage [53]. Our results indicated that SIT treatment upregulates the p62–Keap1–Nrf2 signalling pathway and promotes Nrf2 nuclear translocation in SAP-ALI. Interestingly, in the SAP group, Nrf2 changes at the nuclear level were limited; however, they accumulated in the cytoplasm. Under the action of SIT, Nrf2 migrated to the nucleus and inhibited autophagy, thereby reducing inflammation. Nrf2 regulation is achieved through various pathways, including the Keap1-dependent and Keap1-independent pathways. The latter regulates the Nrf2–ARE pathway mainly through the phosphorylation sites and is considered a critical regulatory factor of Nrf2 nuclear accumulation, nuclear rejection, and degradation [54]. The status and regulatory mechanism of Nrf2 in SAP-ALI are worthy of further research. In mouse lung epithelial-12 cells, Nrf2 silencing significantly decreases nuclear Nrf2 expression and exacerbates oxygen and glucose deprivation/reperfusion-induced autophagy [55]. The role of sitagliptin may be closely related to the nuclear translocation of Nrf2. Furthermore, Nrf2 knockout may reduce Nrf2 entry into the nucleus and fail to initiate the transcription of downstream molecules, thereby exacerbating inflammation and autophagy. Sitagliptin can also reduce ROS production, which mainly depends on the p62–Keap1–Nrf2 signalling pathway.

We designed an in vitro experiment to verify the relationship among ROS, autophagy, and the p62–Keap1–Nrf2 signalling pathway; the results showed that the levels of autophagy and ROS increased after Nrf2 knockdown in BEAS-2B cells. This finding is consistent with the results of our in vivo experiment. The expression of autophagy-related proteins in the knockout mice increased. Therefore, the Nrf2 pathway is directly related to autophagy. Our results showed that SIT could not inhibit inflammation and excessive autophagy in knockout mice, confirming a direct relationship between SIT and Nrf2. Therefore, we infer that the therapeutic effect of SIT is closely related to the activation of the p62–Keap1–Nrf2 signalling pathway, promotion of Nrf2 nuclear translocation, reduction in the ROS levels, and inhibition of excessive autophagy. We could not determine whether SIT directly affects the Nrf2 pathway and autophagy in the lung with SAP or whether the changes observed are indirect results of improved pancreatic inflammation; this requires further research. Choi et al. [56] reported that after DPP4 siRNA transfection into vascular smooth muscle cells, NQO1 expression increases, whereas Keap1 and p62 expression decreases. Chen et al. [57] found that in human umbilical vein endothelial cells, DPP4 inhibition exerts anti-senescence effects by promoting Nrf2 expression and transportation into the nucleus. In our study, we found DPP4 was upregulated after Nrf2 knockout. Although DPP4 negatively correlated with Nrf2, their regulatory mechanism needs further research.

In summary, we confirmed the significant effect of SIT on the inflammatory response, oxidative stress, and autophagy in a mouse model of SAP-ALI. Our results also indicate that the protective mechanism of SIT is closely related to the p62–Keap1–Nrf2 pathway. In BEAS-2B cells, we verified that inflammation, ROS production, and autophagy increased after Nrf2 knockdown. We conclude that SIT inhibits inflammation, ROS generation, and excessive autophagy by activating the p62–Keap1–Nrf2 pathway in SAP-ALI and promoting Nrf2 nuclear translocation, ultimately playing a protective role. These findings provide a theoretical basis for the clinical application of SIT in SAP treatment and development of treatment strategies involving SIT.

## Supplementary information

collated supplementary material

## Data Availability

All data generated during this study are included either in this article or in the supplementary information files.
