# Supplementary material for: Sitagliptin activates the p62–Keap1–Nrf2 signalling pathway to alleviate oxidative stress and excessive autophagy in severe acute pancreatitis-related acute lung injury
Source: Cell Death Dis. 2021 Oct 11;12(10):928. doi: 10.1038/s41419-021-04227-0 (PMC8505515; doi:10.1038/s41419-021-04227-0)
Supplement: Supplementary file 1 — collated supplementary material [file 41419_2021_4227_MOESM1_ESM.pdf]

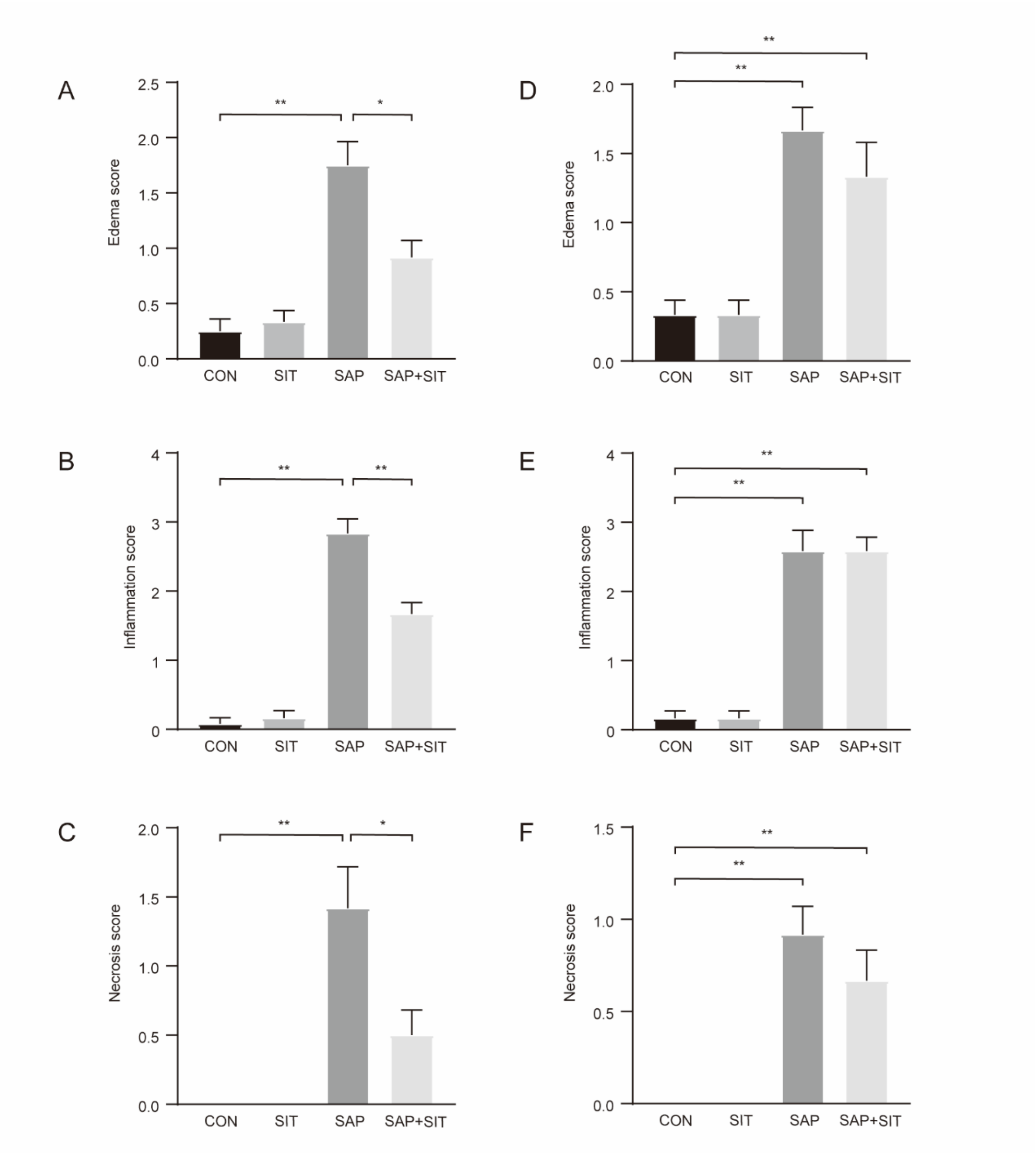

Supplementary Figure 1 Detailed histological scores of pancreatic damage. **a** Edema score of WT mice. **b** Inflammatory score of WT mice. **c** Necrosis score of WT mice. **d** Edema score of Nrf2-/- mice. **e** Inflammatory score of Nrf2-/- mice. **f** Necrosis score of Nrf2-/- mice. *P <0:01 and **P <0:05. Data are presented as mean ± standard error of the mean (SEM) (n =6).

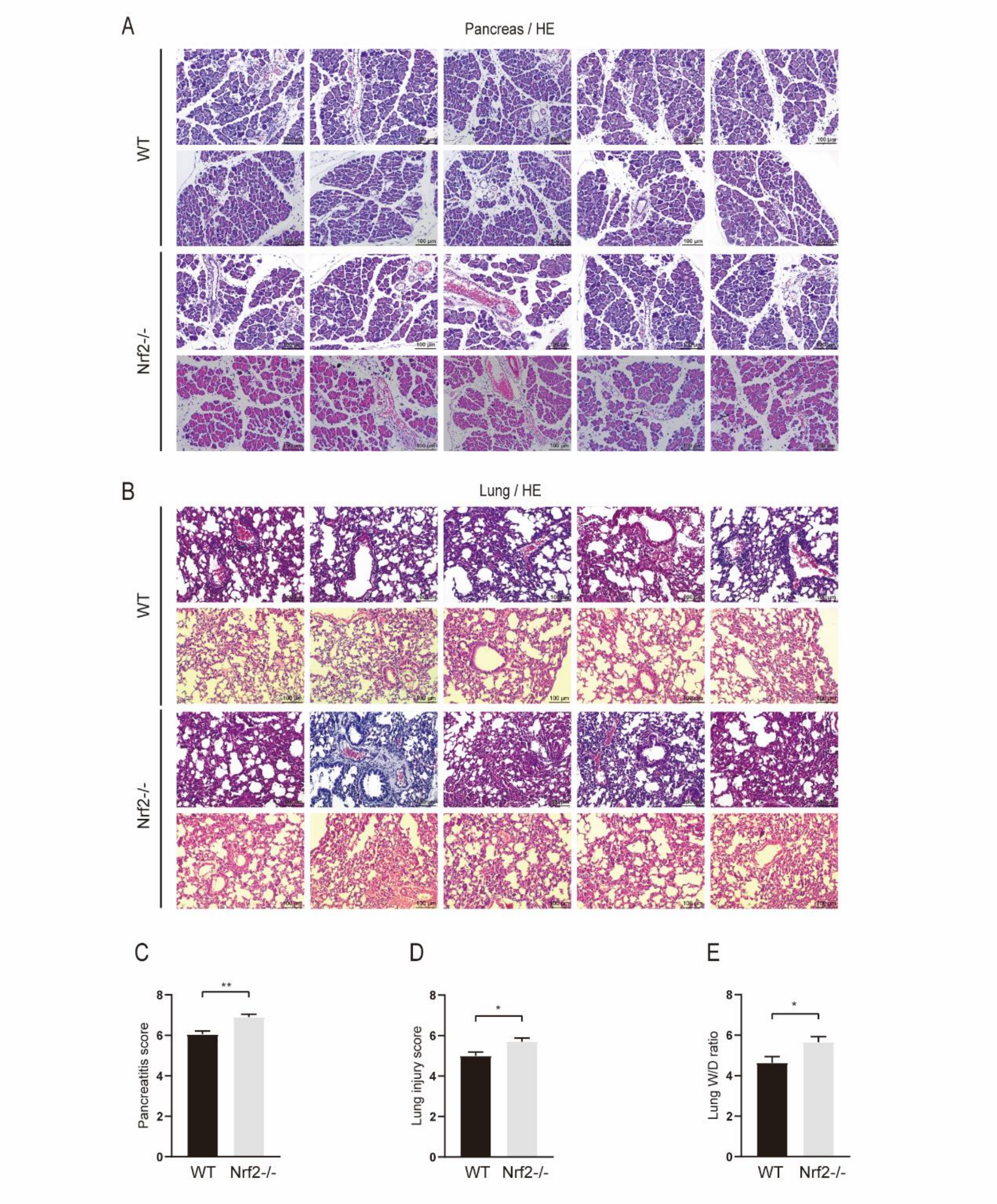

Supplementary Figure 2 Nrf2-knockout mice had more severe inflammation. **a** Representative H&E staining of pancreas tissue (×200). **b** Representative H&E staining of lung tissue (×200). **c** The pancreatitis score. **d** The lung injury score. **e** The lung W/D ratio. *P <0:01 and **P <0:05. Data are presented as mean ± standard error of the mean (SEM) (n =10).

麦杰生物基因鉴定报告

MJ0726-47

送检人	部门	品系编号	品系名称	项目类型	品系背景	样品类型
沈洁	代理繁育	J017009	Nfe2l2	KO	B6J	鼠尾

样品编号	样品数量	配繁信息	繁育代数
1---10	10	mut/wt 配 mut/mut 或<br>mut/mut 配 mut/mut	N10+PN1F9+N<br>1F8

测序信息	阳性对照	鉴定人	鉴定日期

备注

引物

S071	13444	GCCTGAGAGCTGTAGGCCC	Wt： 262 bp	
S072	13445	GGAATGGAAAATAGCTCCTGCC		
S071	13444	GCCTGAGAGCTGTAGGCCC	Mut：~400bp	
S073	oIMR8495	GACAGTATCGGCCTCAGGAA		

PCR 体系

**Reaction Components**	**Volume （μL）**
gDNA Template	2.0
10×*Taq* Buffer(mg^{2+} plus)	2.0
dNTP Mixture（10 mM）	0.5
Primer mix（10 μM）	0.5
Taq DNA polymerase(5 U/μL)	0.5
Milli-Q H_2O	To 20μL

PCR 程序

**Seg.**	**Temp.**	**Time**	**Cycle**
1	95ºC	5min	
2	95 ºC	30s	
3	60ºC	30s	
4	72 ºC	30s	2-4,40
5	72 ºC	3min	
6	25 ºC	hold	

P2 引物加倍

**Seg.**	**Temp.**	**Time**	**Cycle**
1	95ºC	5min	
2	95 ºC	30s	
3	65ºC	30s	-0.5 ºC
4	72 ºC	30s	2-4, 20
5	95 ºC	30s	
6	60 ºC	30s	
7	72 ºC	30s	5-7, 20
8	72 ºC	3min	
9	25 ºC	hold	

P1 65-60TH

凝胶浓度 **1.5%**

麦杰生物基因鉴定报告

<table>
<tr><td>电泳图</td><td>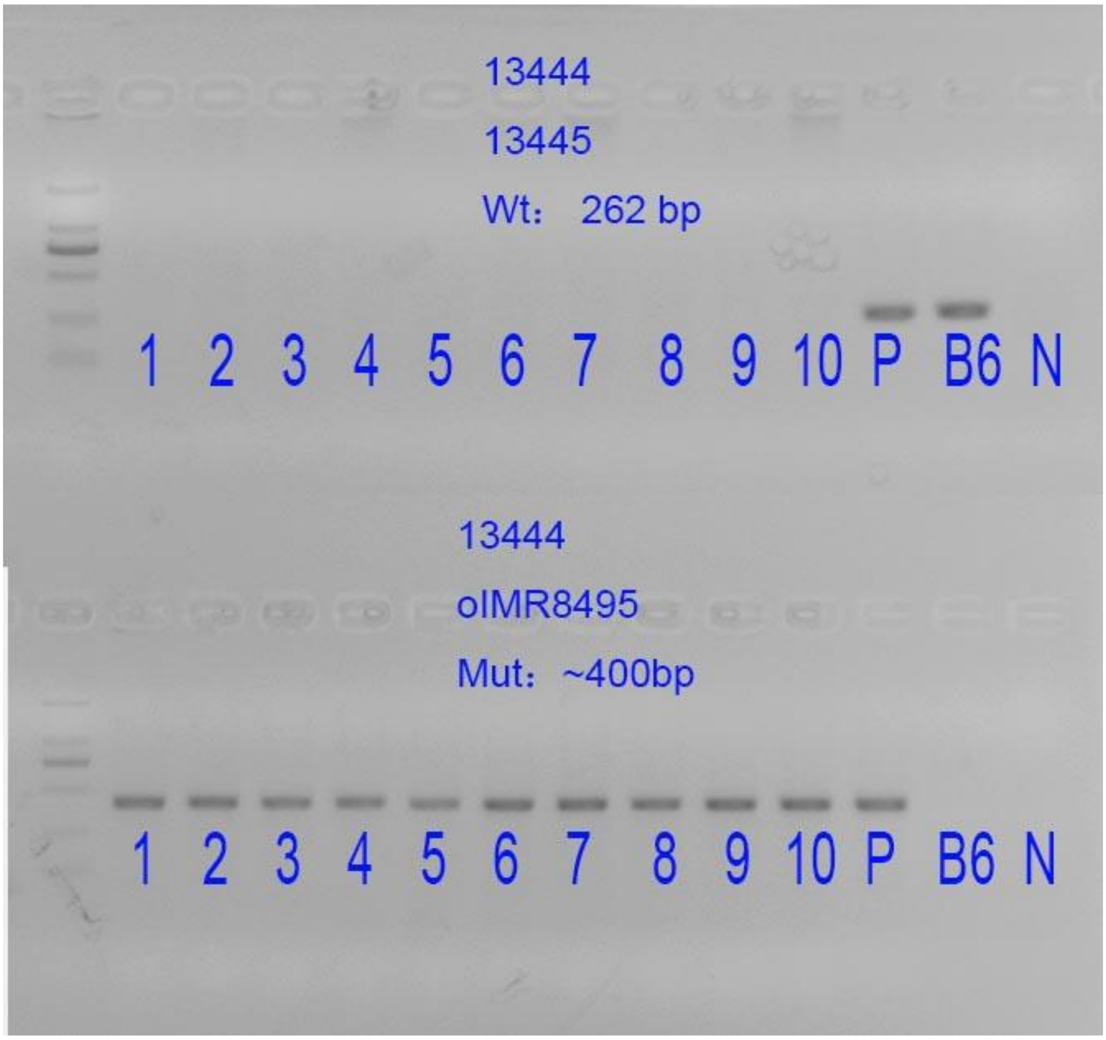

P: Positive Control，为阳性对照
B6：Negative Control 为阴性对照，采用 B6 小鼠基因组 DNA
N：No-Template Control 为无模板的对照
DL2000 Marker：2000bp\1000bp\750bp\500bp\250bp\100bp</td></tr>
<tr><td>结果判定</td><td>B6.129X1-Nfe2l2tm1ywk/J
Mut/Mut：全部</td></tr>
<tr><td>其他</td><td></td></tr>
</table>
